# High-intensity transcranial alternating current stimulation combined with pharmacotherapy for adolescent major depressive disorder: a prospective case report study

**DOI:** 10.3389/fpsyt.2025.1664698

**Published:** 2025-10-01

**Authors:** Ziyi Yuan, Ying Hu, Dandan Cheng, Yilin Yang, Su Hong, Li Kuang

**Affiliations:** ^1^ Mental Health Center, University-Town Hospital of Chongqing Medical University, Chongqing, China; ^2^ Department of Psychiatric, The First Affiliated Hospital of Chongqing Medical University, Chongqing, China; ^3^ Psychiatric Center, The First Affiliated Hospital of Chongqing Medical University, Chongqing, China

**Keywords:** high-intensity transcranial alternating current stimulation, adolescent major depressive disorder, non-invasive brain stimulation, prospective case series study, suicide intervention

## Abstract

**Background:**

Adolescent major depressive disorder (MDD) presents a growing public health concern due to the limited efficacy of current treatments, while high-intensity transcranial alternating current stimulation (HI-tACS) has shown promise in adults but remains unstudied in adolescents. This study therefore examined the preliminary efficacy and safety of HI-tACS combined with pharmacotherapy for first-episode adolescent MDD, with a primary focus on suicide risk reduction.

**Methods:**

In this prospective case series, seven adolescents (aged 12–17 years) diagnosed with MDD received a 4-week intervention combining HI-tACS (77.5 Hz/15 mA, once daily for 20 sessions) with pharmacotherapy. All participants received a standardized pharmacological regimen consisting of sertraline hydrochloride with adjunctive aripiprazole, while oxazepam was permitted as needed for insomnia. Clinical outcomes were assessed at baseline, week 2, and week 4 using the 24-item Hamilton Depression Rating Scale (HAMD-24) and the Patient Health Questionnaire (PHQ-9).

**Results:**

After 4 weeks of treatment, 57.1% of patients achieved a clinical response (≥ 50% reduction in HAMD-24 total score) and 14.3% reached remission (HAMD-24 total score ≤ 8). The HAMD-24 total score significantly decreased at Weeks 2 (Z = –2.366, p = 0.018) and Week 4 (Z = –2.196, p = 0.028). PHQ-9 total scores showed more pronounced improvement during the early treatment phase. Suicide risk initially declined but then rose slightly in the later phase. The intervention was well tolerated with no serious adverse events reported.

**Conclusions:**

The combination of HI-tACS and pharmacotherapy demonstrated potential early effects in this small cohort of adolescents with MDD, particularly during the initial phase of treatment. These preliminary findings warrant further investigation through large-scale randomized controlled trials to establish efficacy and safety profiles, and to further characterize suicide risk trajectories.

## 1 Introduction

Major depressive disorder (MDD) represents a significant global mental health challenge, particularly among adolescents who constitute a highly vulnerable population. Epidemiological studies reveal concerning prevalence rates, including depressive symptoms in approximately 30% of Chinese junior high school students and a global prevalence of mental disorders (including MDD) of 14.3% among adolescents aged 10–19 years (1). Compared to adults, adolescent MDD patients exhibit greater emotional lability and higher rates of impulsive behaviors such as self-harm and suicide attempts (2). These manifestations contribute to substantial disability and mortality, imposing a significant disease burden (3).

The optimal treatment strategy for adolescent MDD remains an active area of research. Current evidence strongly supports the superior efficacy of combined neuromodulation and pharmacotherapy approaches, which achieve synergistic effects through multi-target mechanisms (4, 5). These interventions simultaneously modulate neurotransmitter homeostasis (6), promote neural circuit remodeling (7), enhance neuroplasticity (34), and optimize functional connectivity (9). Such multimodal interventions demonstrate particular advantages in accelerating symptom relief (10), mitigating suicide risk (11), reducing medication-related adverse effects (12), and improving long-term outcomes (13).

Among emerging noninvasive brain stimulation (NIBS) technologies, high-intensity transcranial alternating current stimulation (HI-tACS) has emerged as a potential intervention. This modality delivers biphasic sinusoidal currents at specific frequencies (alpha, gamma, theta) with peak intensities of 15 mA, allowing precise modulation of deep limbic structures (e.g., hippocampus, insula, amygdala) and enhanced global neural synchrony (14, 15). Compared to conventional NIBS, HI-tACS retains the ability to modulate endogenous neural oscillations and induce synaptic plasticity (16), while addressing limitations such as low-intensity stimulation and limited spatial coverage. Clinical trials in adults have demonstrated that 20 sessions of 77.5Hz, 15mA HI-tACS over four weeks - administered as monotherapy or combined with pharmacotherapy - yields significant therapeutic benefits with a favorable safety and tolerability profile (17).

Notably, the adolescent brain’s unique developmental characteristics may confer greater sensitivity to HI-tACS intervention, including: active synaptic pruning and neural circuit reorganization (18), enhanced neural stem cell proliferative capacity (19), and amplified environmental sensitivity - collectively facilitating enhanced neuroplasticity compared to adults (20). Specifically, adolescents exhibit lower functional integration of the default mode network (DMN), facilitating prefrontal connectivity modulation (21) and potentially enhancing HI-tACS efficacy against negative rumination (22). Furthermore, the imbalanced glutamate-γ-aminobutyric acid (GABA) homeostasis in the adolescent prefrontal cortex, while contributing to impaired emotional regulation (23), maybe simultaneously creates a unique therapeutic window for tACS to reduce suicide risk by rebalancing excitation-inhibition dynamics and remodeling neural circuits.

However, these adolescent-specific neurophysiological characteristics reflect intrinsic instability of the developing brain, potentially increasing interventional risks related to adults. Given that HI-tACS at 15 mA exceeds conventional NIBS intensities (< 2 mA), its application in adolescents demands extreme caution. Notably, HI-tACS demonstrates a safety profile superior to that of conventional NIBS in adults, with fewer adverse effects (24). Building upon established clinical experience with NIBS and electroconvulsive therapy in adolescents (25), we propose that HI-tACS warrants systematic investigation as a potential intervention for adolescent depression. Accordingly, we conducted a pilot phase prior to large-scale trials. Due to ethical considerations, high-risk suicide cases were excluded; however, patients with mild suicidal risk without active suicidal behavior were included, reflecting the high comorbidity of suicidal ideation and behavior in adolescent MDD (26).

This prospective case-series study has two primary objectives: (1) to conduct preliminary evaluation of HI-tACS combined with pharmacotherapy in treatment-naïve adolescents with first-episode MDD, and (2) to explore its potential effects on suicide risk. These findings will provide an empirical foundation for future large-scale HI-tACS studies in adolescent populations.

## 2 Methods

### 2.1 Study design and ethics

As the first application of HI-tACS in adolescents MDD, we prioritized establishing initial feasibility data in this vulnerable population. This study was conducted at the First Affiliated Hospital of Chongqing Medical University from October to December 2023. The protocol received ethical approval from the Ethics Committee of this hospital (Approval No.: 2023-299) and was formally registered with the Chinese Clinical Trial Registry (Registration No.: ChiCTR2400084251) and the Medical Research Registration & Filing System (Record ID: MR-50-24-026685). Inclusion of a control group was deemed ethically untenable at this stage due to: (a) the population vulnerability, (b) the absence of prior adolescent HI-tACS safety data, and (c) insufficient statistical power for between-group comparisons.

All potential participants and their legal guardians received comprehensive informed about the study, including its objectives, intervention procedures, potential risks, and benefits. Participation was entirely voluntary, and retained the right to withdraw at any time without compromising their routine clinical care. All enrolled participants and their guardians signed written informed consent forms. Continuous medical monitoring and follow-up observations were provided throughout the study period.

### 2.2 Participants

First-episode, treatment-naive adolescent patients with major depressive disorder (MDD) were recruited from the Psychiatry Outpatient Clinic of the First Affiliated Hospital of Chongqing Medical University.

Inclusion criteria: (1) first-episode depression patients aged 12–17 years; (2) meeting the Diagnostic and Statistical Manual of Mental Disorders, Fifth Edition (DSM-5) criteria for MDD, confirmed by two psychiatrists via the Mini International Neuropsychiatric Interview for Children and Adolescents (MINI-KID); (3) the 24-item Hamilton Depression Scale (HAMD-24) score ≥ 21 and the item-3 of HAMD-24 (suicidality) ≤ 2; (4) no prior exposure to psychiatric medications or physical therapies; (5) capable of understanding questionnaire content and providing truthful responses; (6) voluntary participation with written informed consent provided by legal guardians.

Exclusion criteria: (1) schizophrenia, bipolar disorder, obsessive compulsive disorder, anorexia nervosa, bulimia nervosa, and any axis II disorders (borderline personality disorder, antisocial personality disorder, schizotypal personality disorder and narcissistic personality disorder); (2) organic mental disorders or severe physical illnesses; (3) comorbid other mental disorders (assessed by MINI-KID); (4) history of severe suicidal behavior; (5) substance abuse or dependence; (6) impaired skin integrity at electrode placement sites; (7) implanted electronic devices; (8) unable to cooperate with completing assessments.

Termination criteria: (1) withdrawal requested by the patient or their guardian; (2) occurrence of severe adverse reactions; (3) clinical deterioration or emergence of suicidal behavior; (4) development of other diseases affecting outcome assessment; (5) diagnostic changes due to disease progression.

### 2.3 tACS intervention

The Nexalin^®^ ADI transcranial microcurrent stimulation device (Nexalin Technology, Inc., Houston, TX, USA) was used. The device was equipped with three electrodes: a frontotemporal main electrode (4.45×9.53 cm) placed at Fpz, Fp1, and Fp2 sites according to the 10/20 system, and bilateral mastoid electrodes (3.18×3.81 cm). The treatment protocol consisted of 4 consecutive weeks of daily sessions (Monday to Friday), with a 40-minute duration per session. The parameters were set at 77.5 Hz frequency and 15 mA current intensity. During treatment, patients were instructed to remain relaxed and avoid using electronic devices.

The intervention was performed by professionally trained technicians in strict accordance with the operation manual of the Nexalin^®^ ADI device. Skin integrity was carefully checked before and after each treatment session. During the procedure, patients’ vital signs were monitored, and they were closely questioned about any discomfort.

During the intervention, medication was standardized for all participants with sertraline hydrochloride – with an initial dose of 25 mg/d and titrated to 100 mg/d within one week and maintained thereafter. Aripiprazole was used as adjunctive therapy at a dose of 2.5 mg/d (27). Oxazepam (7.5–15 mg) was administered as needed based on patients’ sleep status. Medication dosages were individually adjusted according to patients’ clinical response and tolerance, as detailed in **Table 1**.

**Table 1:** Clinical characteristics and outcomes of study participants.

	Patient							Group [Mean(SD)]
	1	2	3	4	5	6	7	
Clinical indicators								
Sex	M	M	F	F	F	M	M	
Age	17	13	15	13	16	16	16	15.14 (1.57) (1.46)
BMI	16.9	24.9	20.2	20.2	18.8	21.9	26.66	21.37 (3.39) (3.14)
Duration of MDD (months)	12	2	24	12	6	6	1	9.00 (7.31)
Total score of HAMD-24								
Baseline	27	21	30	36	25	33	27	29.29 (3.65) (4.65)
Week2	20	9*	15*	24	20	34	17	19.86 (7.24) (7.24)
Week4	8*	4*	14*	24	14	28	13*	15.00 (7.80) (7.80)
Total score of PHQ-9								
Baseline	16	1	20	20	8	21	15	14.43 (6.86) (6.86)
Week2	12	0	13	18	7	22	12	12.00 (6.62) (6.61)
Week4	3	0	11	19	4	23	12	10.29 (7.92) (7.92)
Item 3 on HAMD-24								
Baseline	0	2	1	2	2	0	2	1.29 (0.88)
Week2	0	0	0	2	0	0	1	0.43 (0.73)
Week4	0	0	0	2	0	0	2	0.57 (0.88)
Item 9 on PHQ-9								
Baseline	0	0	1	3	0	0	1	0.71 (1.03)
Week2	0	0	0	2	0	2	0	0.57 (0.88)
Week4	0	0	0	3	0	1	1	0.71 (1.03)
Antidepressants type (mg)								
Sertraline hydrochloride	100	100	100	100	100	100	100	100 (0.00)
Aripiprazole	2.5	2.5	2.5	2.5	2.5	2.5	2.5	2.50 (0.00)
Oxazepam	0	0	7.5	7.5	7.5	15	7.5	6.43 (4.79)

F, female; M, male; HAMD-24,24-item Hamiton Rating Scale for Depression; PHQ-9, Patient Health Questionnaire-9.

*Response defined as HAMD-24 score≥50% decrease from the baseline.

### 2.4 Efficacy and safety assessment

Efficacy assessment was conducted in a blinded manner by two consistency-trained raters at baseline, after 10 sessions of HI-tACS intervention (Week 2), and after 20 sessions of HI-tACS intervention (Week 4). The HAMD-24 and Patient Health Questionnaire-9 (PHQ-9) were used to jointly evaluate the efficacy of participants. The primary outcome was the HAMD-24 score reduction rate, defined as the percentage decrease in HAMD-24 total score from baseline to Week 4 [HAMD-24 reduction rate at Week 4 = (baseline score - Week 4 score)/baseline score × 100%]. We defined HAMD-24 reduction rate > 50% as clinical response and HAMD-24 total score< 8 as “clinical remission”. The secondary outcome included: (1) changes in HAMD-24 total score at week 2; (2) changes in PHQ-9 total score; (3) suicide risk indicators: the scores of item 3 on HAMD-24 and item 9 on PHQ-9.

Adverse event was assessed before and after each intervention using the structured checklist of the Common Terminology Criteria for Adverse Events (CTCAE) v5.0 (28), categorizing the adverse symptoms into Grade 1 to Grade 5 according to the clinical manifestation.

All the assessment was conducted by trained assessors strictly following the standards. All results are documented in Case Report Forms (CRFs) and reviewed by independent monitors to ensure consistency.

### 2.5 Data analysis

Statistical analyses were performed using IBM SPSS Statistics Version 26.0. Continuous variables were described using mean and standard deviation (SD), while categorical variables were described using percentages. Paired-samples Wilcoxon rank sum tests were employed to determine significant changes from baseline to Week 2 and Week 4. The significance level was set at α = 0.05 (two-tailed).

## 3 Results

### 3.1 Patient characteristics

**Figure 1:**
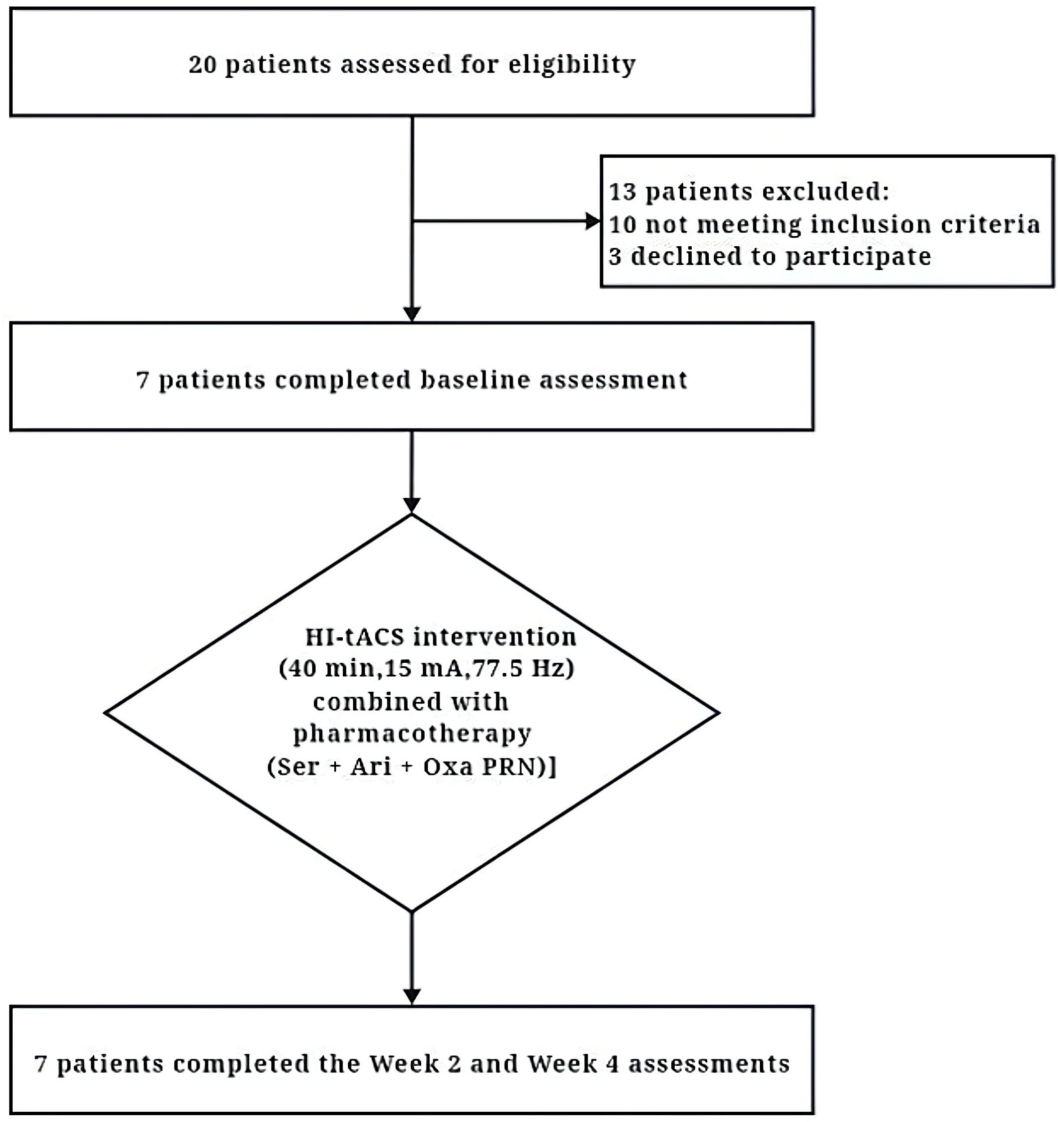
The CONSORT (Consolidated Standards of Reporting Trials) diagram of the primary phases of the clinical trial. HI-tACS, High-Intensity transcranial alternating current stimulation; Ser, Sertraline; Ari, Aripiprazole; Oxa, Oxazepam; PRN, Pro re nata (as needed).

A total of 20 potential participants were screened. Following strict screening against the selection criteria, 7 adolescent patients who met the inclusion criteria were enrolled and completed all 20 sessions of intervention (**Figure 1**). All enrolled patients underwent a comprehensive physical examination at baseline, including assessments of vital signs (blood pressure, heart rate, respiratory rate, and body temperature), height, weight, and neurological examinations (consciousness status, pupillary reflexes, muscle strength, reflexes, and sensory function). No abnormalities were detected in these examinations. Baseline characteristics presented a male-to-female ratio of 4:3 and a mean (SD) age of 15.14 (1.46) years. The mean disease duration was 9.00 (7.31) months and the mean (SD) body mass index (BMI) was within the normal range at 21.40 (3.14) kg/m². Demographic and clinical characteristics are presented in **Table 1**. Notably, Participant 7 presented had comorbid metabolic abnormalities (hyperlipidemia and hyperglycemia) and a history of urethral dilatation. Participants 4 and 6 reported inadequate family support systems. Participant 4 additionally had a history of recurrent self-harm behaviors. Participants 6 and 7 exhibited prominent somatic symptoms. The remaining participants primarily reported academic-related environmental stressors, with no significant physical comorbidities, adequate family support, and otherwise stable medical histories. Regarding patient perspectives, only Participant 6 exhibited initial resistance. The caregiver of Participant 4 reported neutral attitudes, citing dissatisfaction with treatment efficacy. All other participants and their caregivers expressed satisfaction with both the treatment process and outcomes.

### 3.2 Clinical outcomes

**Figure 2:**
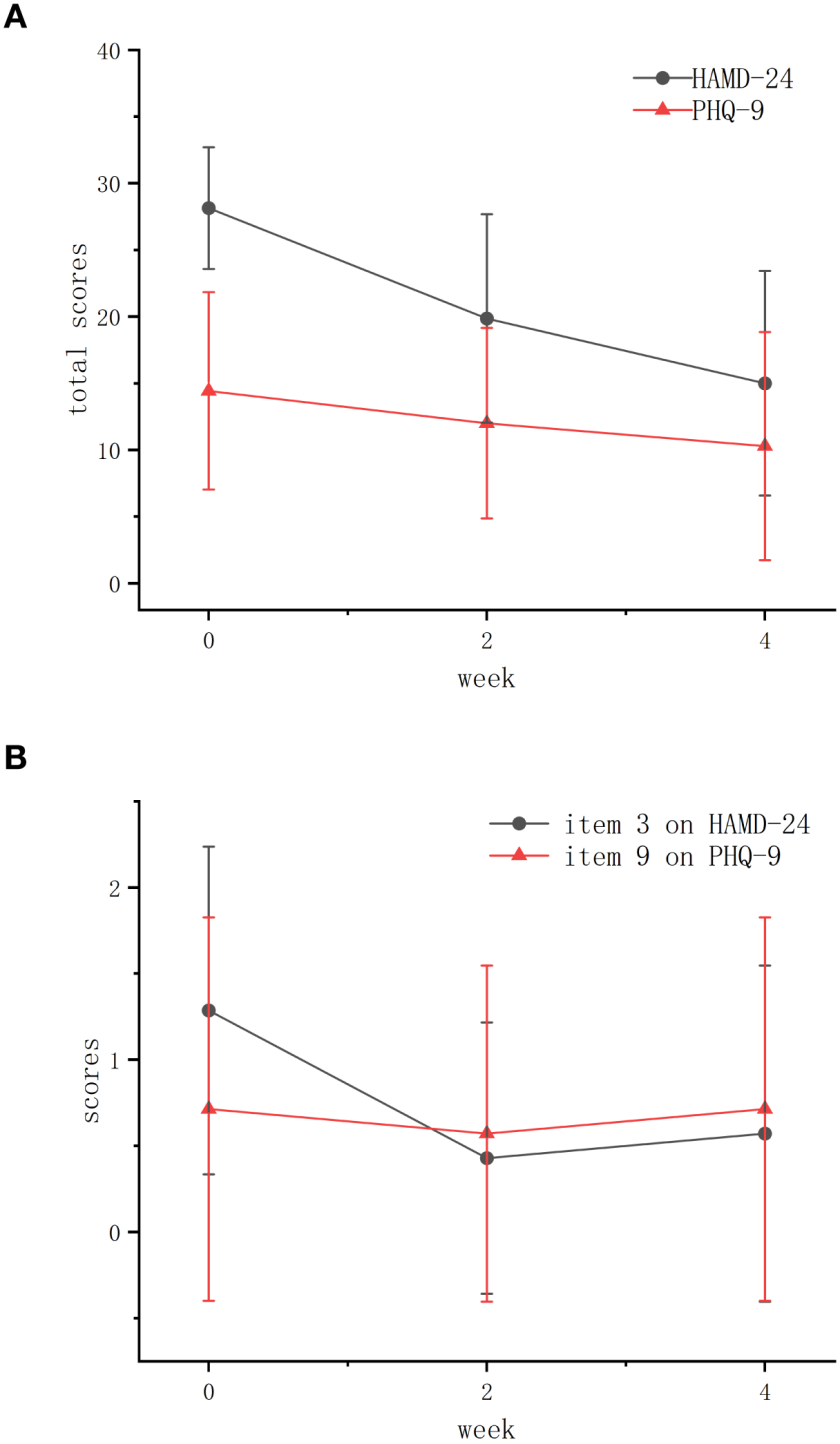
The changes of average total scores of HAMD-24 and PHQ-9 at baseline, week 2 and week 4. **(A)** The average total scores of both scales. **(B)** The average scores of item 3 on HAMD-24 and item 9 on PHQ-9. HAMD-24, the 24-item Hamilton Depression Scale; PHQ-9, the Patient Health Questionnaire-9.

The HAMD-24 total score demonstrated a mean reduction of 13.4 points (95% CI [-20.1, -6.8]) and a median reduction of 13.5 points (95% bootstrap CI [-18.2, -7.1]) from baseline to week 4. Response and remission rates were 57.1% (95% CI [18.4%, 90.1%]) and 14.3% (95% CI [0.4%, 57.9%]) respectively. Paired-samples Wilcoxon rank sum tests revealed a consistent improvement in HAMD-24 total scores, with scores significantly decreasing from baseline to Week 2 (Z = -2.366, p = 0.018) and Week 4 (Z = -2.196, p = 0.028). The reduction from Week 2 to Week 4 was also significant (Z = -2.207, p = 0.027) (**Table 1**; **Figure 2A**).

PHQ-9 scores also showed significant improvement from baseline to Week 2 (Z = -2.043, p = 0.041). Changes between Week 2–Week 4 (Z = -1.219, p = 0.223) and baseline–Week 4 (Z = -1.863, p = 0.063) were non-significant, though the peak effect size (r = 0.70) suggests clinical relevance (**Table 1**; **Figure 2A**).

Analysis of suicide-related items showed that the mean (SD) score of item 3 on HAMD-24 decreased at Week 2 [from 1.29 (0.88) to 0.43 (0.73)], with a slight rebound at Week 4 [from 0.43 (0.73) to 0.57 (0.88)]. The mean (SD) score of item 9 on PHQ-9 decreased at Week 2 [from 0.71 (1.03) to 0.57 (0.88)] before returning to baseline levels at Week 4 [from 0.57 (0.88) to 0.71 (1.03)] (**Table 1**). These trends indicate initial suicide risk reduction of these 7 participants during early treatment, with subsequent fluctuations in later phase (**Figure 2B**).

### 3.3 Safety assessment

Throughout the intervention period, all patients maintained stable vital signs. No new abnormalities were detected in repeated physical or neurological examinations (including consciousness, pupillary reflexes, muscle strength, and sensory function). Furthermore, no severe adverse events (e.g., seizures or altered consciousness) occurred. Only Participant 7 reported scalp pruritus under the Fp1 electrode (CTCAE Grade 1) during the 15th treatment session, which resolved promptly after electrode adjustment, without affecting subsequent treatment. The adverse event rate (14.3%) was comparable to adult HI-tACS studies (29), confirming good tolerability in this adolescent cohort. Furthermore, the combined pharmacotherapy regimen was well tolerated, with no treatment discontinuation due to adverse reactions.

## 4 Discussion

This study represents the first clinical investigation evaluating the therapeutic potential of HI-tACS (77.5 Hz, 15 mA) combined with pharmacotherapy in treatment-naïve adolescents with first-episode MDD. Although efficacy metrics appear modest compared to adult studies reporting response rates of 70% and remission rates of 60% (17), they nevertheless provide preliminary evidence for the therapeutic potential of HI-tACS in adolescents. These results warrant the conduct of larger-scale randomized controlled trials to systematically evaluate HI-tACS’s efficacy and safety in adolescent MDD.

Longitudinal treatment responses revealed distinct temporal dynamics. During the early treatment phase (Weeks 1-2), statistically significant reductions occurred in both HAMD-24 and PHQ-9 total scores (p< 0.05), including improvement in core depressive symptoms (HAMD-24 item 3) and suicidal risk (PHQ-9 item 9). This early response pattern suggests that HI-tACS may rapidly alleviate acute depressive symptoms and mitigate suicide risk initially. However, therapeutic trajectory diverged in later phases (Weeks 3-4): HAMD-24 scores continued to decline at a reduced rate, while PHQ-9 scores failed to maintain statistical significance, accompanied by modest rebound in suicide risk metrics. This pattern aligns with prior observations in adults receiving theta burst stimulation (iTBS/cTBS) for treatment-resistant depression, indicating potential shared mechanism across neuromodulation therapies (30). Based on these clinical and mechanistic evidence, we propose that HI-tACS exerts biphasic effects in adolescent MDD: early-phase responses likely reflect immediate neuromodulation, including α-wave asymmetry normalization (31), θ-wave synchronization enhancement (32), and prefrontal GABAergic circuit regulation (33), whereas later-phase effects involve gradual monoamine homeostasis restoration (34) and structural neuroplastic reorganization (16) to sustain therapeutic benefits. Further validation through extended follow-up, multimodal neuroimaging, and translational research is warranted to elucidate these mechanisms.

Notably, our analysis revealed substantial interindividual variability in treatment response among the 7 participants. Among the 4 responders (Participants 1, 2, 3, and 7), complete resolution of suicidal ideation was achieved in 3 cases (excluding Participants 7). Among the 3 non-responders (Participants 4, 5, and 6), Participants 5 demonstrated clinically relevant improvement with marked reduction in suicidal risk despite on not meeting formal response criteria (44% HAMD-24 reduction). Parent-mandated school attendance despite patient’s severe school refusal tendency constituted the primary barrier to clinical response in this case. Conversely, another two non-responders (Participants 4 and 6) both exhibited converging psychosocial vulnerabilities: Participant 4 experienced prolonged parental absence during development, correlating with maladaptive emotion regulation manifesting as recurrent self-injury. Participant 6 came from an economically disadvantaged background with a disabled father and a low-education mother as sole provider, creating persistent psychological stressors. A rebound in suicidal risk was observed in Participants 4, 6 and 7, warranting clinical attention. The insufficient family support systems of Participants 4 and 6 suggest that external psychosocial stressors and underdeveloped adaptive coping strategies likely contributed to the rebound suicidal risk (35). In the case of Participant 7, the rebound in suicidal risk may be underpinned by distinct neurobiological mechanisms secondary to his metabolic comorbidities. Dyslipidemia is a potent driver of neuroinflammation, oxidative stress, and impaired mitochondrial function within the central nervous system. These processes can disrupt prefrontal-limbic circuitry, diminish neurotrophic support, and ultimately compromise the brain’s ability to regulate emotion and impulsive behavior—thereby escalating suicide risk (36). Other neurophysiological factors may also contribute to the rebound. Specifically, neural adaptation to repeated stimulation (16) potentially diminished anti-suicidal efficacy over time, while incompletely consolidated brain network connectivity (9) may have left the nascent DMN vulnerable to relapse and the suicidal risk to resurface. Future large-scale studies should systematically evaluate these potential moderators to enable personalized intervention strategies.

Regarding safety, all 7 adolescent participants demonstrated good tolerability to 77.5Hz/15mA HI-tACS without severe adverse events (e.g., seizures, impaired consciousness). Only one case (14.3%) reported mild transient scalp pruritus, aligning with adult HI-tACS studies, supporting continued use of these parameters in larger adolescent trails.

## 5 Limitations

This preliminary study suggests HI-tACS combined with pharmacotherapy may provide rapid symptom relief in adolescents MDD. Larger controlled trials are needed to confirm efficacy, establish safety, optimize parameters, and develop predictive models for personalized application.

However, several limitations warrant acknowledgment in this study. First, the small sample size (n=7) and lack of suicide-specific assessment tools limited statistical power and precise evaluation of anti-suicidal efficacy. Second, the open-label design without medication-only or sham controls cannot exclude concomitant medication or placebo effects. Third, the absence of follow-up assessments precluded long-term efficacy evaluation. Finally, the wide confidence intervals from limited sampling reduced effect size precision.

To address these constraints, a large-scale follow-up study with a more rigorous methodology, including a control group and extended follow-up, is currently in progress. Future research should focus on: multimodal neuroimaging (fMRI/EEG) and biomarker analysis to clarify HI-tACS mechanisms; triple-blind, sham-controlled RCTs; larger cohorts with longitudinal follow-up to assess sustained efficacy; optimization of stimulation parameters (frequency, intensity, duration) for personalized protocols; and exploration of combined treatment approaches with rTMS, ECT or CBT, to enhance treatment outcomes.

## Data Availability

The original contributions presented in the study are included in the article/supplementary material. Further inquiries can be directed to the corresponding authors.
